# The effect of probiotic cheese consumption on inflammatory and anti-inflammatory markers, disease severity, and symptoms in patients with rheumatoid arthritis: study protocol for a randomized, double-blind, placebo-controlled trial

**DOI:** 10.1186/s13063-022-06113-2

**Published:** 2022-02-24

**Authors:** Farzaneh Asoudeh, Kurosh Djafarian, Maassoumeh Akhalghi, Mahdi Mahmoudi, Ahmad Reza Jamshidi, Elham Farhadi, Ahmad Esmaillzadeh

**Affiliations:** 1 Department of Clinical Nutrition, School of Nutritional Sciences and Dietetics, Tehran University of Medical Sciences, Tehran, Iran; 2 Rheumatology Research Center, Tehran University of Medical Sciences, Tehran, Iran; 3 Department of Community Nutrition, School of Nutritional Sciences and Dietetics, Tehran University of Medical Sciences, P.O. Box 14155-6117, Tehran, Iran; 4 Obesity and Eating Habits Research Center, Endocrinology and Metabolism Molecular-Cellular Sciences Institute, Tehran University of Medical Sciences, Tehran, Iran; 5 Department of Community Nutrition, School of Nutrition and Food Science, Isfahan University of Medical Sciences, Isfahan, Iran

**Keywords:** Probiotics, Probiotic cheese, Rheumatoid arthritis

## Abstract

**Background:**

In recent decades, several studies have shown changes in the intestinal microflora among patients with rheumatoid arthritis (RA). Therapeutic measures using probiotics have shown favorable effects on the recovery of these patients. However, most studies have used probiotic supplements. In this study, we aimed to investigate the effect of probiotic cheese consumption on inflammatory and anti-inflammatory factors, disease severity, and symptoms in these patients.

**Methods:**

This study is a randomized, double-blind clinical trial, in which forty patients with mild to moderate severity of RA will be randomly allocated to receive either 30 g/day probiotic cheese (*n* = 20) or only low-salt and low-fat cheese without any added probiotic (*n* = 20) for 12 weeks. Assessment of anthropometric measures and biochemical indicators, including serum concentrations of high-sensitivity C-reactive protein (hs-CRP), tumor necrosis factor-α (TNF-α), interleukin-6 (IL-6), and interleukin-10 (IL-10), will be done at the study baseline and end of the trial. In addition, disease severity and disability will be assessed by DAS-28 and the HAQ-DI questionnaire, respectively.

**Discussion:**

Diet is the leading environmental factor affecting the gut microbiota. A prebiotic-rich diet and probiotics might be beneficial in this regard. To the best of our knowledge, the effect of probiotic supplements on inflammation in these patients has widely been assessed; however, there is only one study that examined the effect of probiotic-containing food in these patients. Further studies are needed to investigate the effect of probiotic-containing foods on inflammatory markers and symptoms in patients with RA.

**Trial registration:**

Iranian Registry of Clinical Trials IRCT20201120049449N1. Registered on 14 February 2021

## Background

Rheumatoid arthritis (RA) is an inflammatory autoimmune condition that can lead to cartilage and bone destruction and eventually joint deformities. It affects 0.5 to 1% of the world’s population [1] and is more common in women than in men [2]. The prevalence of this condition in Iranian adults was 0.33% [3].

Several factors are involved in the onset and progression of RA, but the exact physiopathology is yet unknown. In addition to genetics, several environmental factors, including diet, smoking, alcohol consumption, have been studied in this regard. Nutritional interventions along with medications, physiotherapy, and regular exercise have been applied in the management of RA [4–7]. The imbalanced intestinal microbiota has also recently gained significant attention in this regard. The disturbance in the gut microbiota might alter the immune function through elevating pro-inflammatory factors, which can, in turn, exacerbate the severity of RA [8–10]. Numerous interventions have used probiotic supplements in the prevention and treatment of RA. Findings from these investigations have revealed a favorable effect of probiotic supplements on inflammatory and anti-inflammatory cytokines in these patients [11, 12].

However, probiotic supplementation, in which a high dose of probiotics is given to patients, might endanger the patients’ health [13]. Widespread and unregulated administration of probiotics, especially in persons who have a weak immune system, can result in infection, sepsis, metabolic disturbances, and immunological defects [13]. In addition, probiotic supplements are expensive, and given that most RA patients have low socioeconomic status and expend on other medications as well, administration of probiotic supplements to these patients would impose a further cost to these individuals. Therefore, it is essential to find an appropriate approach to carry probiotics into the body without these problems. Earlier studies have shown that probiotic-rich foods are effective in cooling down inflammation as strong as supplements [14–16]. Consumption of probiotic foods has several benefits, including taking probiotics in more controlled dosages. In addition, maintaining the consumption of these foods in routine life is more accessible than taking probiotic supplements for a loge time. Despite several studies about the effect of probiotic supplementation on inflammatory symptoms in patients with RA, we are aware of only one study that examined the effect of probiotic foods in these patients. In a clinical trial conducted on patients with RA, Nenonem et al. examined the effect of uncooked Lactobacilli-rich fermented wheat drink for 8 or 12 weeks. They found that this probiotic diet decrease symptoms of rheumatoid arthritis. However, the dose of probiotics in these foods was uncertain in that study, and they chose an uncommon food to carry probiotics into the body. Furthermore, they examined the effect of this intervention on high-sensitivity C-reactive protein (hs-CRP) only [17]. Therefore, this randomized, placebo-controlled, parallel clinical trial was designed to investigate the effect of probiotic cheese consumption on inflammatory, anti-inflammatory, disease activity, and severity factors in patients with rheumatoid arthritis.

## Methods/design

### Participants

**Fig. 1:**
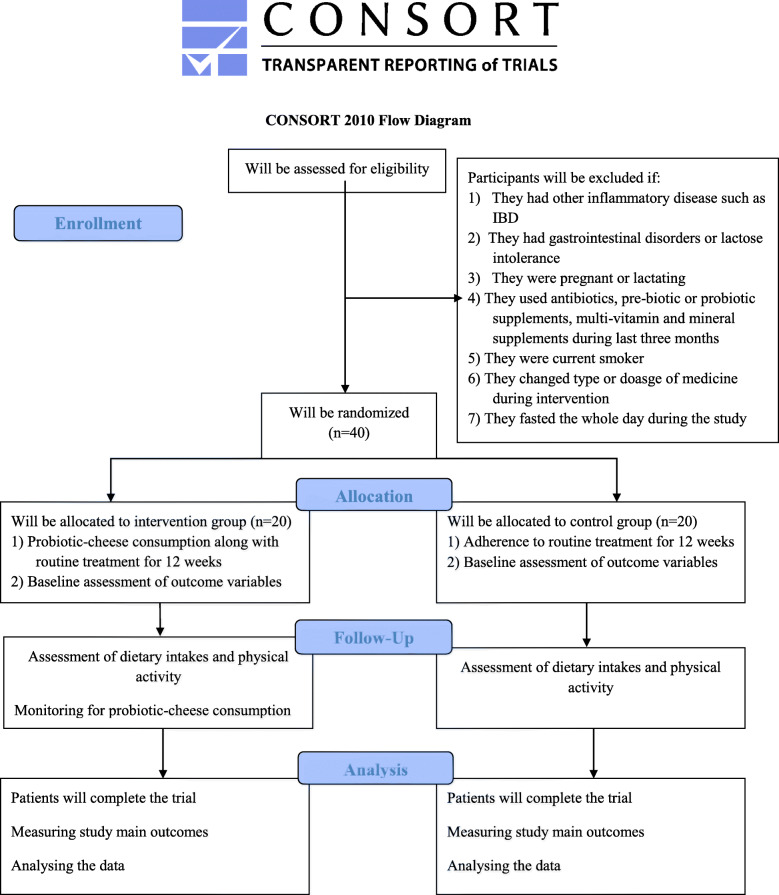
Diagram of the study design

This study is a parallel superiority randomized clinical trial, with a 1:1 allocation ratio which will be done in 2021, in which participants will be recruited from Shariati Hospital, Tehran, Iran. Women who will meet the criteria will be included in the current study: (1) those aged 20–60 years, (2) have a body mass index (BMI) of 25–40 kg/m^2^, and (3) those who have been diagnosed with a mild to moderate RA. RA will be diagnosed by a rheumatologist using the American College of Rheumatology (ACR) criteria. Disease Activity Score-28 (DAS-28) > 3.2 will be considered as mild to moderate RA [18, 19]. Patients whose conventional medications (methotrexate, prednisolone, hydroxychloroquine, sulfasalazine) were not changed in terms of type or dosage over the past month will be included. Individuals with (1) other inflammatory diseases including pancreatitis and inflammatory bowel disease (IBD), (2) acute heart disease or kidney and liver disease, (3) gastrointestinal disorders or lactose intolerance, (4) those who are pregnant or lactating, and (5) those who were using antibiotics, pre-biotic or probiotic supplements, multi-vitamin, and mineral supplements during the last 3 months will not be included. The study would be restricted to non-smokers and non-alcoholic individuals. People who are routinely using probiotic-enriched foods in their usual diet will not be included as well. The exclusion criteria would be any alteration in the type or dosage of medicines during the intervention, taking dietary supplements or antibiotics, smoking and alcohol use throughout the study, and getting pregnant. We will also exclude patients who fasted the whole day during the study, as Muslims do during Ramadan. All participants will provide informed written consent. Moreover, the study protocol has been recorded on the IRCT website (IRCT20201120049449N1). The flow diagram of the project is shown in Fig. 1.

### Study design and intervention

**Fig. 2:**
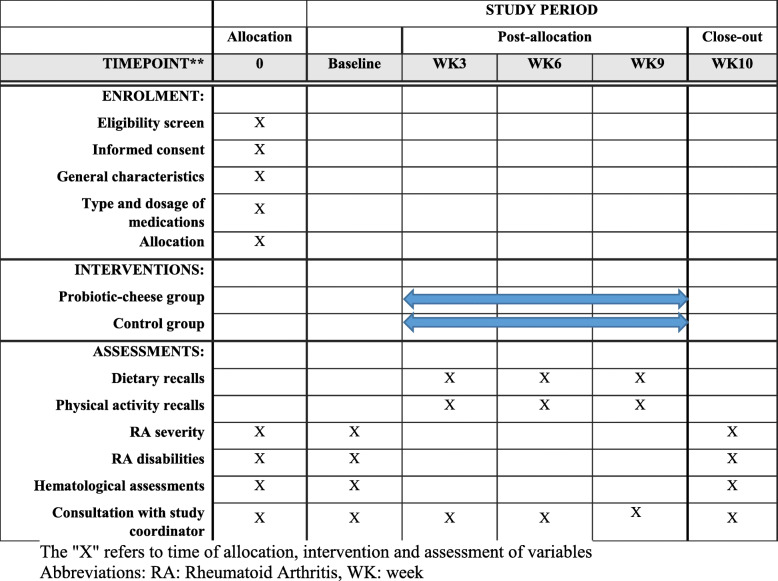
Standard Protocol Items: Recommendations for Interventional Trials (SPIRIT) chart of the enrollments and assessments during randomized controlled trials

The diagram for Standard Protocol Items: Recommendations for Interventional Trials (SPIRIT) is shown in Fig. 2. After recruiting participants, they will be stratified based on age (20 to < 40 and 40–60 years), BMI (< 30 and ≥ 30 kg/m^2^), and type of medicines (nonsteroidal anti-inflammatory drugs (NSAIDs)/disease-modifying antirheumatic drugs (DMARDs)/steroids/immunosuppressive) into different blocks and then will be randomly allocated to the intervention or control groups. For each patient in a particular block, a matched person in terms of the abovementioned variables would be placed in that block. Then, these two patients in a given block would be randomly assigned to the intervention and control groups. To do randomization, an identification code will be given to each eligible patient in each block, and then, the code of patients with the same age, BMI, and medication will be stated in the lottery container. A person who will be unaware of the study aims will be asked to draw the codes out of the container randomly. The first code will be assigned to the intervention group and the second code to the control group. This will be done for all containers. All patients, researchers, rheumatologists, statistical analysts, and laboratory staff will be blind to the intervention. Random allocation will be done by a third person who is unaware of the aim of our study. Patients in the intervention group (*n* = 20) will receive 30 g/day low-salt and low-fat probiotic cheese. Participants in the control group (*n* = 20) will receive low-salt and low-fat cheese without probiotics. Packaging of both kinds of cheeses would be in the same shape and color. The intervention will last for 12 weeks. Assessment of primary and secondary outcome variables will be done at the study baseline and end of the trial. A standard container will be given to participants, and they will be asked to that container to make sure they are consuming the specified amount. Participants will be asked not to change their lifestyle, dietary habits, and medicines throughout the study. To assess dietary intakes throughout the study, we will fill four 1-day 24-h dietary recalls (including two working days and two non-working days) once every 3 weeks by telephone interview. To complete dietary recalls, participants will be asked to report dietary intakes based on household measures. Then, we will convert the reported amounts to grams using available booklets. The average dietary intakes in these 3 days will be considered as participants’ mean dietary intakes throughout the intervention. To obtain nutrient intakes based on these food recalls, we will use the Nutritionist IV software (based on US National Nutrient Databank) modified for Iranian foods. In addition, four 1-day physical activity records (including two working days and two holidays) will be completed from all patients at weeks 3, 6, 9, and 12 of intervention to assess physical activity during the study. Participants will be requested to report all activities they have during a specific day with the name and type of activity as well as the time spent. All participants will be educated on how to record their activities throughout the study before the start of the trial. Furthermore, patients in both groups will be asked not to change their physical activity throughout the trial.

### Adherence

To determine the adherence to the intervention, individuals will be asked to record their daily consumption of cheese in a checklist given to them by the investigators. To increase compliance and avoid forgetting the use of cheese, participants will receive short messages on their cellphones every day as reminders. We also ask patients to deliver empty packages of cheese.

### Adverse events

We will ask the patients to report any adverse events that may have occurred during the study. These events will be recorded by investigators. If a given trial participant will complain about any rare harmful effect of the intervention or will want to quit the study based on personal reasons, we will exclude her/him from our trial.

## Study outcomes

The main primary outcome in this study would be the evaluation of serum concentrations of high-sensitivity C-reactive protein (hs-CRP). We will also consider the assessment of other inflammatory factors, including tumor necrosis factor-α (TNF-α), interleukin 6 (IL-6), and anti-inflammatory factor interleukin-10 (IL-10) as well as the assessment of disease severity and disease-related disabilities lipid profile, anthropometric measures, dietary intake, and physical activity, which all would be considered as secondary outcomes.

### Blood sampling and biochemical measurements

To assess blood factors, 10 cc blood sampling will be taken from all patients after 12 h of overnight fasting. The Westergren method will be used to measure the erythrocyte sedimentation rate (ESR). Previous studies have shown that this index is highly correlated with the severity of RA disease. In order to measure inflammatory factors, after separating the serum from the blood sample, the serums will be stored in a − 80 °C freezer until the tests are performed. Serum levels of inflammatory factors including hs-CRP, TNF-α, IL-6, and anti-inflammatory factor IL-10 will be measured by enzyme-linked immunosorbent assay (ELISA) method using the commercial kits. Serum concentrations of these indicators were strongly correlated with disease severity among patients with RA.

### Assessment of disease severity

Disease severity will be assessed using a valid DAS-28 scale [20]. The scale, designed by Prevoo et al. in 1995, evaluates 28 joints and consists of 4 components: (1) counting the number of swollen joints, (2) counting the number of painful joints, (3) ESR or hs-CRP levels, and (4) Global Health Score (Global Health) determined by visual analog scale (VAS) [20]. The total scores of the whole scale will vary from 0 to 9.4, and based on that score, patients will be classified into four categories: (A) individuals with a score of more than 5.1 would be called as those with a high disease activity, (B) those with a score between 3.2 and 5.1 will be defined as individuals with moderate disease activity, (C) patients with a score between 3.2 and 2.6 will be defined as individuals with low disease activity, and finally, (D) those with a score of less than 2.6 will be considered in remission stage (recovery) [18, 19]. This tool has previously been used in Iranian patients with rheumatoid arthritis, and the results have been acceptable [21–24].

### Assessment of disease related-disabilities

To examine the disease-related disabilities, we will apply the Health Assessment Questionnaire-Disability Index (HAQ-DI). Health Assessment Questionnaire (HAQ) was designed in 1978 by James F. Fries et al. at Stanford University and included five subscales [25]. One of its subscales is HAQ-DI which has been used as an independent questionnaire in several studies [25]. This scale consists of 20 questions in 8 categories, and the range of scoring for each question varies from 0 to 3 (greater scores demonstrate more significant disability). To compute the overall HAQ-DI score, we used the average of eight categories’ scores. The total score of 0 to 1 will be considered mild to moderate disability, 1 to 2 as moderate to severe disability, and 2 to 3 as severe to very severe disability. We will not calculate the HAQ-DI score when a patient will not provide answers for more than two categories [25].

### Assessment of anthropometric measures

We will obtain data on anthropometric indices, including body weight, height, and BMI at the study baseline and end of the trial. Bodyweight will be measured in a fasting state, without shoes while wearing light clothing to the nearest 0.1 kg, using a weighing calibrated scale (Seca, Hamburg, Germany). Height will be measured by a mounted tape, without shoes and at a standing position to the nearest 0.1 cm using a stadiometer (Seca, Hamburg, Germany). BMI will be calculated by dividing weight in kilograms by height in meters squared.

### Assessment of dietary intake and physical activity

To ensure adherence to the intervention and avoid changes in dietary intakes throughout the study, assessment of dietary intake will be done using four dietary records throughout the study, one in every 3 weeks. Participants will be trained at the study baseline on how to record their dietary intakes. Days of dietary recording will be determined by investigators, such that two weekdays and two weekend days will be included in these recordings. Subjects will be requested not to change their usual dietary intakes on the day of recording their intakes. All records will be reviewed immediately, and possible problems will be resolved by interview. To compute nutrient intakes of study participants based on the analysis of these dietary records, we will use the Nutritionist IV software, in which the original database is based on the United States Department of Agriculture (USDA) food composition table; however, a modification will be done for the Iranian local foods.

To ensure a lack of difference in activity levels between intervention and non-intervention groups throughout the study, physical activity will be assessed by asking study participants to record their usual activities on specific days. The number of physical activity records will also be four, including two weekdays and two weekends. All the records will be taken once in 2 weeks. Therefore, all patients will complete four dietary records and four physical activity records during the study. Data from physical activity records will be processed using MET-h/day values given for each physical activity based on published guidelines [26].

### Study sample size calculation

The required sample size was calculated based on the data from a previous study [27] considering a type I error of 5% (*α* = 0.05) and type II error of 20% (*β* = 0.20, power = 80%) and hs-CRP as the key variable. We considered the mean difference in serum hs-CRP levels of 5.71 between the two groups. Also, the average SDs reported for hs-CRP in the control and intervention groups were considered as 5.93. We used the suggested formula for parallel clinical trials and reached the sample size of 17 patients in each group.

$$ n=\frac{2\times {\left({Z}_{1-\frac{\alpha }{2}}+{Z}_{1-\beta}\right)}^2\times \mathrm{SD}2}{\mathrm{d}{}^2} $$

$$ n=2\times {\left(1.96+0.85\right)}^2{(5.93)}^2/{(5.71)}^2=17 $$

Based on this formula and given a 20% drop-out in each group, we will need a sample size of 20 persons for each group.

### Data management

We will store participants’ information according to TUMS rules. Participant data will be anonymized to keep privacy, by ID numbers in all records. In addition, we will separately store all identifying participants’ characteristics in a password-protected secure study database. Only trial researchers will be allowed access to the login passwords. When data collection was finished, we will destroy the participant’s identification data and the participant’s consent forms. Information related to evaluation protocols will be stored up to 5 years in the same database. All biological specimens will be stored in the biochemistry lab of TUMS. The coding, security, and storage of data will be checked by the principal investigator (AE). Data assessment will be conducted electronically by a co-investigator (FA). Moreover, AE will examine the data entry and data values two times.

### Statistical analysis

Statistical analyses of all data will be performed using the SPSS software version 21.0 (SPSS Inc., Chicago, IL, USA). To handle non-adherence, we will use the intention-to-treat approach for data analysis. Missing values will be dealt with using the last observation carried forward (LOCF) method. Data will be expressed as mean ± standard deviations (± SD) and percentages. The one-sample Kolmogorov–Smirnov test will be used for assessing the normality of the distribution of data. In the case of a non-normal distribution of a variable, we will apply the log transformation. If the variables were non-normally distributed even after log transformation, we would use non-parametric tests in the analyses. Continuous variables will be compared between the two groups by independent samples *t*-test (or its non-parametric equivalent). We will apply the chi-square test to examine the distribution of participants in terms of categorical variables. To find the effect of the intervention on outcome variables, repeated measures analysis of variance (or its non-parametric equivalent) will be used. In these analyses, the effect of the intervention, time, and time-intervention interaction will be explored. The potential difference in baseline levels of outcome variables as well as differences in dietary intakes and physical activity levels, if any, between the two groups will be controlled for in these analyses. *P* values will be considered significant if they were < 0.05.

### Interim analyses

We do not anticipate interim analysis or stopping rules in our current study due to the intervention’s properties. There is no adverse effect of probiotic cheese consumption in earlier studies.

### Plans to give access to the full protocol, participant-level data, and statistical code

The complete details of the study protocol can be accessed via IRCT.ir (IRCT20201120049449N1). Any participants’ related data and datasets analyzed during the study will be available by contacting the corresponding author.

### Oversight and monitoring

FA, KDj, and AE, who conceived the study design, will be the lead study coordinators of the study. The Trial Steering Committee (TSC) are FA, KDj, and AE. In addition, administrative and research advisors (MM, AJ), laboratory advisor (EF), and study physician (MA) will also help do the study. The committee will convene every month in order to oversee the progression of the study and manage the financial or technical problems. There is no need for a separate Data Monitoring Committee due to the low-risk nature of the study. The coordinating centers are the clinical nutrition department, biochemistry lab, and rheumatology research center at Tehran University of Medical Sciences. All laboratory tests, including blood sampling, serum collection, and specimen storage, will be conducted in the biochemistry lab. FA is responsible for coordinating patients’ visits and collecting the consent forms. The Data Management Team will consist of the project investigators (FA, KDJ, and AE). At the study end, in order to appreciate the participants, all patients will be consulted by a nutritionist, and a specific meal plan for her/him will be provided.

### Frequency and plans for auditing trial conduct

We have not considered any plan for auditing trial conduct. Moreover, FA is responsible for informing the Trial Steering Committee and Ethics Committee about any unpredicted risks throughout the study period.

### Plans for communicating important protocol amendments to relevant parties (e.g., trial participants, ethical committees)

Whenever any major amendments which may impress the conduct of the trial or patient safety will be needed, we will communicate the Trial Steering Committee and all participants. Also, we will document any deviation from the trial protocol using a breach report form. An edited version of the study protocol will be published on the Iranian Registry for Clinical Trials (IRCT.ir).

## Discussion

RA is a chronic disease that is associated with a wide range of comorbid conditions [28]. There is no definitive treatment for RA. Common medications such as corticosteroids and NSAIDs have various complications like increased risk of cardiovascular disease and infection [29]. Patients with RA have significant alterations in gut microbiota composition [30]. Also, the gut microbiota in RA patients showed lower diversity, and there was a relationship between low diversity of gut microbiota and increased disease duration [31]. Intestinal dysbiosis can influence the intestinal barrier integrity and function. Diet is the main environmental factor affecting the gut microbiota [32]. A prebiotic-rich diet and probiotics might be beneficial in this regard [33]. Some studies have considered probiotic consumption plausible adjuvant therapy for RA patients [34, 35]. Probiotics, through their direct and indirect immune system modulation, regulate inflammatory status and disease severity in these patients [36]. To the best of our knowledge, there is only one study that examined the effect of probiotic-containing food in these patients [17]; however, the effect of probiotic supplements on inflammation in these patients has widely been assessed [21–24]. Assessment of the probiotic-containing foods might provide further insight into the effect of probiotics in these patients because probiotic supplements might not be affordable in all individuals. We choose cheese as a carrier for probiotics in this study because earlier studies have introduced dairy products as the best carrier for probiotics. Among dairy products, cheese consumption is much more usual than other products in these patients, as we found in our pilot study before the inception of the current trial. The finding of the current study cannot be used to recommend to the general population the use of probiotics. However, they might be used in clinical settings by dietitians to recommend (or not recommend) RA patients to include probiotic-enriched foods in their diets. Further clinical trials with a large sample size are needed to support our findings.

## Strengths and limitations

This is the first clinical trial investigating the effects of probiotic cheese consumption on inflammatory and anti-inflammatory markers, disease severity, and symptoms in patients with RA. It should be noted that the intervention is of low cost and can easily be carried out in clinical practice. In this study, patients will be block-matched with each other in terms of several variables that might influence the findings. Several outcomes, including biochemical indicators along with subjective- and objective-based symptoms, will be examined in patients at the study baseline and end of the trial. Moreover, we will assess compliance with the intervention throughout the study. We hypothesize that probiotic cheese consumption might result in decreased inflammation and improvement in the gut microbiota, which might lead to beneficial effects on disease severity and symptoms.

The limited number of bacterial species in the probiotic cheese is the main limitation of the current project. Due to limitations in budget, we would not be able to evaluate a wide range of inflammatory and anti-inflammatory cytokines. To examine the compliance of study participants to the prescribed probiotic-containing food, one might suggest to assess trimethylamine N-oxide (TMAO); however, the lack of funding to do this would be another limitation. In addition, subjective decisions in the assessment of outcome variables might also be of concern.

## Data Availability

Not applicable.

## Abbreviations

RA: Rheumatoid arthritis
BMI: Body mass index
ACR: American College of Rheumatology
DAS-28: Disease Activity Score-28
IBD: Inflammatory bowel disease
SPIRIT: Standard Protocol Items Recommendations for Interventional Trials
NSAIDs: Nonsteroidal anti-inflammatory drugs
DMARDs: Disease-modifying antirheumatic drugs
ESR: Erythrocyte sedimentation rate
hs-CRP: High-sensitivity C-reactive protein
TNF-α: Tumor necrosis factor-α
IL-6: Interleukin 6
IL-10: Interleukin-10
ELISA: Enzyme-linked immunosorbent assay
VAS: Visual analog scale
HAQ: Health Assessment Questionnaire
HAQ-DI: Health Assessment Questionnaire-Disability Index
USDA: United States Department of Agriculture
TMAO: Trimethylamine N-oxide
